# Training for mental health professionals in responding to experienced and anticipated mental health-related discrimination (READ-MH): protocol for an international multisite feasibility study

**DOI:** 10.1186/s40814-022-01208-8

**Published:** 2022-12-13

**Authors:** Claire Henderson, Uta Ouali, Ioannis Bakolis, Nada Berbeche, Kalpana Bhattarai, Elaine Brohan, Anish Cherian, Eshetu Girma, Petra C. Gronholm, Dristy Gurung, Charlotte Hanlon, Sudha Kallakuri, Amanpreet Kaur, Bezawit Ketema, Heidi Lempp, Jie Li, Santosh Loganathan, Pallab K. Maulik, Gurucharan Mendon, Tesfahun Mulatu, Ning Ma, Renee Romeo, Rahul Kodihalli Venkatesh, Yosra Zgueb, Wufang Zhang, Graham Thornicroft

**Affiliations:** 1 Centre for Implementation Science, Health Services and Population Research Department, Institute of Psychiatry, Psychology and Neuroscience, King’s College London, De Crespigny Park Box, London, SE5 8AF UK; 2 South London and Maudsley NHS Foundation Trust, Denmark Hill, London, SE5 8AZ UK; 3 Department Psychiatry A, Razi University Hospital, Cité des Orangers, 2010 La Manouba, Tunisia; 4 Department of Biostatistics and Health Informatics, Institute of Psychiatry, Psychology and Neuroscience, King’s College London, London, UK; 5 Department of Psychology, Laboratory of Clinical Psychology: Intersubjectivity and Culture, University of Tunis, Tunis, Tunisia; 6 Transcultural Psychosocial Organization (TPO), Kathmandu, Nepal; 7 Centre for Global Mental Health, Health Services and Population Research Department, Institute of Psychiatry, Psychology and Neuroscience, King’s College London, De Crespigny Park, London, SE5 8AF UK; 8 National Institute of Mental Health and Neurosciences (NIMHANS), Bengaluru, India; 9 Department of Preventive Medicine, School of Public Health, College of Health Sciences, Addis Ababa University, Addis Ababa, Ethiopia; 10 Centre for Global Mental Health and Centre for Implementation Science, Health Service and Population Research Department, Institute of Psychiatry, Psychology and Neuroscience, King’s College London, De Crespigny Park Box, London, SE5 8AF UK; 11 Department of Psychiatry, WHO Collaborating Centre for Mental Health Research and Capacity-building, School of Medicine, College of Health Sciences, Addis Ababa University, Addis Ababa, Ethiopia; 12 George Institute for Global Health, 308 Elegance Tower, New Delhi, 110025 India; 13 Centre for Rheumatic Diseases, Department of Inflammation Biology, School of Immunology and Microbial Sciences, Faculty of Life Sciences & Medicine, Weston Education Centre, 10, Cutcombe Rd, London, SE5 9RJ UK; 14 The Affiliated Brain Hospital of Guangzhou Medical University, No. 36 Mingxin Road, Liwan District, Guangzhou, 510370 China; 15 Faculty of Medicine, University of New South Wales, Sydney, Australia; 16 Peking University Sixth Hospital (Institute of Mental Health), National Clinical Research Center for Mental Disorders & Key Laboratory of Mental Health, Ministry of Health (Peking University, No 51, Huayuanbei Road, Haidian District, Beijing, 100191 China; 17 King’s Health Economics, Health Service and Population Research Department, Institute of Psychiatry, Psychology and Neuroscience, King’s College London, De Crespigny Park Box, London, SE5 8AF UK; 18 Faculty of Medicine of Tunis, University of Tunis El Manar, Tunis, Tunisia

**Keywords:** Stigma, Discrimination, Training, Health professionals, Mental health care, Objective structured Clinical examination, Health advocacy

## Abstract

**Background:**

Mental health and other health professionals working in mental health care may contribute to the experiences of stigma and discrimination among mental health service users but can also help reduce the impact of stigma on service users. However, few studies of interventions to equip such professionals to be anti-stigma agents took place in high-income countries. This study assesses the feasibility, potential effectiveness and costs of Responding to Experienced and Anticipated Discrimination training for health professionals working in mental health care (READ-MH) across low- and middle-income countries (LMICs).

**Methods:**

This is an uncontrolled pre-post mixed methods feasibility study of READ-MH training at seven sites across five LMICs (China, Ethiopia, India, Nepal and Tunisia). Outcome measures: knowledge based on course content, attitudes to working to address the impact of stigma on service users and skills in responding constructively to service users’ reports of discrimination. The training draws upon the evidence bases for stigma reduction, health advocacy and medical education and is tailored to sites through situational analyses. Its content, delivery methods and intensity were agreed upon through a consensus exercise with site research teams. READ-MH will be delivered to health professionals working in mental health care immediately after baseline data collection; outcome measures will be collected post-training and 3 months post-baseline, followed by qualitative data collection analysed using a combined deductive and inductive approach. Fidelity will be rated during the delivery of READ-MH, and data on training costs will be collected. Quantitative data will be assessed using generalised linear mixed models. Qualitative data will be evaluated by thematic analysis to identify feedback about the training methods and content, including the implementability of the knowledge and skills learned. Pooled and site-specific training costs per trainee and per session will be reported.

**Conclusions:**

The training development used a participatory and contextualised approach. Evaluation design strengths include the diversity of settings, the use of mixed methods, the use of a skills-based measure and the knowledge and attitude measures aligned to the target population and training. Limitations are the uncertain generalisability of skills performance to routine care and the impact of COVID-19 restrictions at several sites limiting qualitative data collection for situational analyses.

## Background

In 1998, *The Lancet* published an essay by Norman Sartorius, then President of the World Psychiatric Association, entitled *Stigma: what can psychiatrists do about it?* [1]. Focusing particularly on schizophrenia, he recommended that psychiatrists (1) expand the focus of clinical work beyond symptom reduction to improving quality of life, (2) reflect on and try to improve their own attitudes by updating their clinical knowledge and learning from those using their services and their families about the impact of the illness and of stigma on them, (3) monitor for discrimination and expand their role to include advocacy and (4) learn from others about how stigma and discrimination can be reduced.

Over 20 years later, it is now timely to consider progress against these recommendations. Outcomes other than clinical ones are increasingly used in research and routine practice, and the concept of personal recovery has had a considerable impact on mental health policy and practice in many countries [2]. Furthermore, continuing professional development is included in the requirements for licence renewals and revalidation for many professionals. Reflective practice is used extensively in undergraduate and postgraduate training [3] which in theory provides scope for examining one’s own attitudes to people with mental illness. While the extent to which this occurs is unknown, stigma among health professionals, including mental health professionals, is an increasing focus of research [4].

Although mental health professionals have more knowledge of mental illness compared to health professionals of other specialities, they are nevertheless exposed to negative cultural stereotypes prior to professional education [5]. Indeed they differ little from the general population in terms of desire for social distance but consistently show less socially restrictive attitudes (except regarding coercion into treatment) and are more supportive of the civil rights of people with mental illness [6–8].

The frequency of discriminatory behaviours reported by service users in mental health care settings ranges from 16 to 44% [9],C. [10]. Qualitative research in England found that service users described a lack of support and a tendency towards overprotectiveness by mental health service providers [11], while in Mexico, service users described the cold and distant treatment and a lack of clear explanations provided by psychiatrists, a focus on psychopathology/medication and lack of interest in their personal history [12]. By means of their particular relationship with the service user, mental health care professionals can contribute to exacerbating or mitigating self-stigma [13]. This form of stigma encompasses negative beliefs about the self, based on shame, the acceptance of mental illness stereotypes, a sense of alienation from others and consequent low self-esteem and mood. It prevents people from seeking health care, employment and social opportunities [14].

However, less stigmatisation of patients by more experienced mental health professionals was observed in several surveys [15, 16, 17]. This may be attributed to a better capacity to prevent burnout and the loss of empathy associated with burnout, more observations of personal recovery in patients, more personal or family experience of mental illness and/or more experience in overriding stereotypes [4].

While the evidence above suggests mental health professionals as a target for a stigma reduction intervention, any such intervention need also to take into consideration the mental health professionals’ potential role as an anti-stigma change agent [18]. Previous articles have acknowledged the potential impact that physicians’ advocacy could have in reducing discrimination (Arboleda-Flórez & Stuart, 2012; Thornicroft et al., 2010; Ungar et al., 2016), agreeing professionals could champion anti-stigma efforts, including much-needed structural changes as health care quality improvement and policy change. The extent to which stigma reduces mental health professionals’ ability to provide effective care is obvious to them and can contribute to burnout and demotivation.

At a policy and service level, stigma contributes to restrictive mental health legislations, the poor coverage of mental health education in university curricula for health professionals, and unequal allocation of health resources with low budgets attributed to mental health care compared to physical health care resulting in poor mental health care resources [19], in over-reliance on institutional care and in limited access to physical health care [20]. Mental health professionals notice service users’ barriers to seeking and engaging with treatment, obstacles to rehabilitation due to discrimination in employment and within social networks, reluctance to pursue economic and social opportunities due to the anticipation of discrimination and negative self-evaluation due to internalised stigma [21]. However, there is little evidence that advocacy and effective stigma reduction methods have been incorporated into the role of psychiatrists or other mental health professionals, based on reviews of stigma reduction interventions [22], G. [23]. As a result, how anti-stigma advocacy can be incorporated by mental health professionals is not yet clear. We therefore need to return to Sartorius’s recommendation to learn from others.

In other fields of medicine and especially that of primary care, there is an increasing focus on physicians’ social accountability and advocacy. Across North America, for example, several organisations have expressed a pressing need for advocacy training in medical education [24, 25], Shaws et al., 2017). The Royal College of Physicians and Surgeons of Canada published a CanMEDS Physician Competency Framework, introducing health advocacy as one of the six main competencies which medical education programmes need to address [26]. The role of a health advocate is understood as the skill to ‘determine and understand needs, speak on behalf of others when required and support the mobilisation of resources to effect change’ (Frank, 2015). The CanMEDS competencies have been adopted in a number of other countries including an adaptation to the Ethiopian context (EthioMEDS) for psychiatry training [27], where psychiatrists are likely to be called upon to develop policy, services and training and to join with service users to advocate for better services. However, there is a need to introduce the concept of an anti-stigma agency to other cadres and to combine it with stigma reduction.

A recent systematic review [28] of the feasibility and effectiveness of training in health advocacy, anti-stigma competency or related skills for health professionals retrieved 39 studies, three of which reported interventions for mental health care professionals [21, 29–31]. Programme content varied widely; some covered mainly interpersonal stigma reduction, others focusing on social determinants of health and some included health advocacy at the structural level. The authors found some evidence for their effectiveness; however, it proved difficult to compare the effectiveness across programmes given the wide variety in content, duration, teaching methods and outcome measures. The majority of studies were carried out in high-income countries (HICs); therefore, it is difficult to extrapolate feasibility to low- and middle-income countries (LMICs) where the mix of professionals and hence the roles they carry out is often different to that of HICs. The authors conclude that to maximise its relevance to the communities served, any intervention for mental health care professionals needs to link to the professionals’ roles, be developed following a situational analysis and include local people with lived experience of mental health problems, use of mental health services and mental health-related stigma in the delivery (experts by experience). Training needs to use interactive delivery methods and initial piloting, and evaluation should examine behavioural change.

We therefore designed Responding to Experienced and Anticipated Discrimination training for health professionals working in mental health services (READ-MH). These may be mental health professionals or health professionals who do not have professional training in mental health but are working in mental health services. Furthermore, our target service setting is specialist mental health services rather than primary care or any other setting. READ-MH was developed based on a previous training for medical students [18, 32], the findings of the above review, situational analysis at the seven sites of the INDIGO Partnership programme [33] comprising desk reviews and qualitative interviews and focus groups with stakeholders on mental health-related stigma, and a consensus development exercise regarding the delivery format, content and teaching methods among the INDIGO Partnership research team based on data from the studies included in the systematic reviews. The present study aims to assess the feasibility, potential effectiveness and costs of the READ-MH training at these sites. We define a feasibility study as addressing the questions of whether something can be done, whether we should proceed with it, and if so, how. The key uncertainties with respect to feasibility concerns are acceptability of the intervention and feasibility of outcome data collection.

## Methods/design

This is a pre-post feasibility study at seven LMIC sites using mixed methods. READ-MH will be delivered to health professionals working in mental health care immediately after baseline data collection; the quantitative follow-up data will be collected post-training and at 3 months post-baseline, followed by qualitative data collection. Fidelity will be rated during READ-MH delivery. In addition, data on the costs of the training will be collected. There is no control group as this is not required to address the aims related to feasibility, and the sample size is not designed to determine the effectiveness.

### Setting

All seven sites across five countries (China, Ethiopia, India, Nepal and Tunisia) of the UK Medical Research Council-funded INDIGO-Partnership research group will take part in the study. Given that sites have variable provisions of mental health care and staffing, sample sizes and targeted professionals vary by site (see Table 1).

**Table 1:** Study settings

Site	Location for training	Target professionals	Target sample and size
Beijing, China	Peking University Sixth Hospital and District Mental Health Hospitals, Haidian, Chaoyang, Xicheng District, Beijing	Psychiatry residents, psychiatrists, psychiatric nurses, social workers	30 mental health professionals
Guangzhou, China	Rehabilitation Department, Affiliated Brain Hospital of Guangzhou Medical University, Liwan district, Guangzhou, China	Psychiatrists, psychiatric nurses, rehabilitation therapists, psychotherapists, social workers	20 mental health professionals
Ethiopia	District Health Offices and Primary Health Centres, Sodo, South Sodo, Misrak Meskan and Meskan districts, Butajira City Administration, South Central Ethiopia	Psychiatric nurses and health administrators or managers involved in coordinating and developing mental health care	A total of 28 (5 psychiatric nurses, 5 mental health coordinators from district health offices and 18 health centre heads or health centre focal persons for mental health)
Bengaluru, India	Rehabilitation Departments, National Institute of Mental Health and neurosciences (NIMHANS)-tertiary care centre	Psychiatry residents, trainees from M.Phil. in Psychiatric Social Work/Psychology/M.Sc. Psychiatry nursing/staff who come in regular contact with a person with mental illness (such as instructors)	24 mental health and non-mental health professionals
Delhi (National Capital Region), India	District Hospital in Faridabad, Haryana	Mental health professionals (1 psychiatrist, 1 counsellor, 1 psychiatric nurse and 1 community nurse)	Four trained mental health professionals
Kathmandu, Nepal	Private and government hospitals and medical colleges Pokhara, Gandaki Province	Psychiatry residents, psychiatrists, psychiatric nurses	6–8 psychiatrists, residents and psychiatric nurses
Tunis, Tunisia	Razi University Hospital La Manouba	Psychiatry residents, nurses working in mental health at the hospital	16–20 psychiatry residents and nurses

### Participants and recruitment

Eligible mental health professionals or health professionals will be any staff working currently in the mental health field in patient-facing roles at each site. At some sites, professionals working in mental health care are not trained as mental health professionals but are health professionals assigned to work in mental health, and will hence be eligible. The administrative staff will be excluded as the skills training is not applicable to their role, but no other exclusions will be made based on qualifications or profession type. A participant information sheet will be distributed by the site researchers to eligible professionals at least 24 h before the first session, and written informed consent will be sought at the start of the first session. Individual written consent will be ensured through the availability of multiple members of the research team who will answer individual professionals’ queries. It will be made clear to professionals by the research team and in the participation information sheet that non-participation will not be penalised by the professional’s supervisor. Participants will not be paid to attend the training as it will take place during working hours, but any expenses will be covered. Participants will be asked for contact details that can be used in case they have changed employers by the time of the 3-month follow-up interview. Non-participants will not be eligible to attend the training.

### Measures

#### Outcome measures

Knowledge will be assessed through a structured questionnaire in the form of a quiz based on the content of the training with input from all sites. The quiz will cover (i) knowledge about sources of stigma, (ii) the impact of stigma including in the context of health care and (iii) how mental health professionals can reduce this impact.

Attitudes of mental health professionals to working to address the impact of stigma on service users will be measured through the Attitudes to addressing stigma and discrimination scale (ASTAD). This scale was created by rewording an existing scale, the Short Alcohol and Alcohol Problems Perception Questionnaire (SAAPPQ) [34]. The original SAAPPQ assesses health professionals’ attitudes to working with people with alcohol problems. It has two items contributing to each of five domains (role adequacy, role legitimacy, motivation, work satisfaction and task-specific self-esteem) and two subscales: role security (the sum of role adequacy and role legitimacy) and therapeutic commitment (the sum of motivation, work satisfaction and task-specific self-esteem). In the British validation study, the SAAPPQ showed a good correlation with the extent of postgraduate training in addiction and the frequency at which GPs initiated discussions about alcohol use during consultations [34]. By retaining the questions and changing the wording from working with people with alcohol problems to working to reduce stigma and discrimination, the ASTAD retains the structure of the SAAPPQ.

Behaviour and communication skills will be assessed through an objective-structured clinical examination (OSCE). These are widely used in medical education and comprise scenarios in which the student/trainee interacts with a simulated patient in the presence of an examiner [35]. The scenario for READ-MH comprises a simulated mental health service user reporting experienced and anticipated discrimination and who is faced with a disclosure decision regarding their mental illness, either in the context of marriage or employment. The OSCE scenario was developed in accordance with the INDIGO Partnership-implementing sites to reflect typical interactions/problems of discrimination/stigma at the sites.

Participating simulated service users will be given a briefing sheet to standardise the scenario and their responses to participating professionals’ questions.

The professional will be assessed by both the simulated patient and the observer on (i) their responses aimed to reduce the impact of stigma, including acknowledging the problem, showing an empathic attitude, exploring the patient’s concerns and identifying important considerations to help the patient make a decision informed by their own values and relevant clinical information, and (ii) stigmatising behaviours such as ignoring the simulated patient’s concerns, dismissing/not believing their report of discrimination, endorsing the discriminatory behaviour reported, attributing responses to anticipated discrimination to laziness or incompetence or telling the patient whether to make a disclosure.

Guidance for the OSCE raters and simulated patients for their assessment will be adapted from existing communication skills OSCEs used at King’s College London, the OSCE developed for the study of READ for medical students and from the ENhancing Assessment of Common Therapeutic factors (ENACT) therapist competence training rating scale [36]. This guidance will be transcribed into a standardised marking scheme describing the assessment process to increase reliability and comparability across sites. Marking will reflect the formative nature of the OSCE.

#### Implementation measures

Quantitative process measures will include (i) attendance records for participants and (ii) a fidelity checklist to record the delivery of and participants’ active participation in the sessions. Items on delivery will cover the use of those evidence-based teaching methods chosen. Each item in the checklist will be scored 0 = not achieved, 1 = partially achieved or 2 = achieved in full, with guidance for the anchor points. Qualitative process data will include feedback on the training immediately post-training and feedback on any impact on the professionals and their practice at 3 months.

### Intervention

We describe READ-MH using the TIDieR checklist [37]. The aims of READ-MH are to increase the ability of professionals working in mental health care to:

1. Recognise their own stigma and minimise the effects on patients, carers, students and trainees in health professions and colleagues
2. Identify and respond constructively to patients’ reports of and anticipated discrimination and self-stigma
3. Address interpersonal discrimination and foster advocacy at the individual, family and service levels

The content areas, delivery methods and duration of READ-MH were finalised using a consensus development exercise conducted remotely as a video call in November 2020, in which research team members from all sites participated, with voting choices based on the results of a systematic review of similar interventions for health professionals [28]. The READ-MH manual was then drafted by CH and UO; UO added content to fit the cultural and social specificities of the Tunisia site, following a cultural adaptation matrix. This matrix was established through situation analysis comprising a desk review on mental health-related stigma in the region and qualitative research with local health care staff, service users and carers. This site-specific content was highlighted so that when the manual was sent to the other sites, researchers at each site could replace it with content from their site, again using the cultural adaptation matrix.

The manual describes each module including methods of delivery (role play, facilitated group discussion, testimony from the expert by experience, etc.) and provides a slide pack and gives specific examples of adaptation to each site. The manual also contains guidance on training and supporting the expert by experience, ground rules for the interaction of mental health professionals with the expert by experience and a safety protocol in case the expert by experience requires support during or after the delivery of READ-MH.

READ-MH training will be provided by research team members at each site who are themselves mental health professionals together with experts by experience. Delivery will be to mental health professionals and health professionals working in mental health care in groups of no more than 12 participants, to facilitate active participation. Training will be delivered in five modules, which allow for flexibility across sites: all in one time, shorter or longer duration of training (4 to 8 h). The different modules, their respective content and teaching methods are shown in Table 2. Training will be in person unless COVID-19 restrictions require online training and Internet connections are sufficiently reliable.

**Table 2:** READ-MH training modules

Module	Module title	Content/objectives	Teaching methods
	Introduction	Introduction of participants Introduction of expert by experience Learning objectives, module outline, course delivery methods	Presentation
1	What is stigma and how does it affect mental health care?	Description and explanation of experienced, anticipated, self-stigma and structural stigma How stigma reduces the effectiveness of mental health care	Interactive lecture Brainstorming Group discussion instigated by thought experiment and supported by evidence from the site Testimonial from an expert by experience and/or audiovisual clip
2	Experienced discrimination	How to identify and respond to experienced discrimination as a mental health professional How to help service users to respond to experienced discrimination How to develop stigma resistance in service users	Testimonial of expert by experience Role play and debriefing—group discussion
3	Anticipated discrimination and self-stigma	How to identify and respond to anticipated discrimination as a mental health professional How to help service users to overcome anticipated discrimination and self-stigma	Testimonial of expert by experience Case vignette Role play and debriefing—group discussion
4	Stigmatisation within mental health care	Structural stigma in mental health care Experienced stigma in mental health care (perpetrated by mental health professionals) Factors contributing to stigma perpetrated by mental health professionals	Interactive lecture Including quotes of stigmatising experiences from service users within the mental health system and clinical case illustrations Group discussion
5	What can we do to reduce stigma in mental health care?	Focus on stress and burnout as factors aggravating stigma; self-care for mental health professionals as a means to reduce stigma Communication skills Role of advocacy	Case study, followed by a group discussion supported by evidence from the sites Roleplay

Experts by experience will play an important role in delivering the training. Each site research team will provide training to experts by experience to prepare them to give personal testimony. The training will be based on previous work at one of the sites [38] and on training for Time to Change Champions (https://www.time-to-change.org.uk/take-action/resources-champions). The contribution of the expert by experience to the training will include (i) descriptions of the illness and personal recovery process, (ii) experiences of stigma and discrimination in the community and in mental health care services and (iii) coping with stigma including with its internalisation and its anticipation and with experiences of discrimination and how they were overcome.

It is possible that experts by experience may find that delivering the training acts as a stressor that affects their mental health or that a mental health professional behaves in a way they find upsetting during the training. To minimise this risk, ground rules for mental health professionals will be established. These will include not asking questions used for clinical assessment of the expert by experience’s mental state, not dismissing the expert’s experience and not interrupting them. We will also use a safety protocol for experts by experience informed by the anti-stigma programme for Time to Change England to ensure they are well-supported. This will include avoidance of training sessions directly before times when support is not accessible, such as weekends; debriefing after each session; and a contact number for experts to call a clinical member of the research team if they wish to debrief at a subsequent time.

### Procedures

#### Quantitative data

Baseline data will be collected immediately before the first training session starts (see Table 3). The OSCE will be administered first, to prevent an influence on behaviour during the OSCE of considering responses to the knowledge quiz and ASTAD. Each OSCE will be rated by one of the trainers or another member of the research team. Afterwards, participants will be given the ASTAD and knowledge quiz for self-completion.

**Table 3:** Measures and assessment time points

	Baseline	During training	Immediate follow-up	3-month follow up
Knowledge quiz	√	–	√	√
ASTAD	√	–	√	√
OSCE-rater	√	–	√	–
OCSE-simulated patient	√	–	√	–
Fidelity measure	–	√	–	–
Qualitative feedback	–	–	√	√

The fidelity checklist and attendance record will be completed by a member of the research team at each session other than the researchers delivering the training. Immediate post-training data collection will occur at the end of the training and will follow the same order as for baseline data collection. Results of the two OSCEs will be provided once all immediate post-training follow-up data have been collected. At 3 months from the baseline, participants will be given or sent the knowledge quiz and ASTAD to self-complete and return.

#### Qualitative data

At each site, all participants in each group will be invited to attend a focus group held once they complete the OSCEs and outcome assessments immediately after the training. The topic guide will cover their perceived relevance and usefulness of the training, aspects they thought worked best versus less well, parts of the training that could be improved and barriers/facilitators to application in practice. The researchers delivering the training will feed back on aspects they felt did or did not go well and the extent of participants’ active participation.

At 3 months follow-up, after completion of the outcome assessments, all participants will be invited to take part in focus groups or individual interviews. The topic guide will cover any perceived impact on practice and if relevant education, barriers/facilitators to application in practice and experiences of application to practice/education. The choice of interviews versus focus groups will be made by each site research team. Interviews and focus groups will be audio-recorded and transcribed verbatim by members of the research site teams.

Table 3 gives an overview of all quantitative and qualitative assessments at different time points.

### Health economics

An economic research question concerns costs: what is the cost to introduce READ-MH? Therefore, in this study, we will examine the cost of READ-MH training, including the comparative costs of such an intervention across different geographical sites. All sites will take part in an interview designed to get a description of the training to be implemented on their site and to obtain their best estimates of the resources required. After the interview, an Excel spreadsheet will be sent to each site, requesting the relevant data to facilitate the derivation of site-specific READ-MH training costs.

Data on time spent by various individuals in training will be measured prospectively by the research teams at each site. Attendance and data on time spent at training sessions will also be measured prospectively by the research team at each site.

All costs will be collected in local currencies and converted to US$ using purchasing power parity (PPP) and local currency unit (LCU) exchange rates for the most recent year. Training sessions will be costed for the trainers and experts by experience using per diem payments and will include preparation time for the sessions. Direct training-related expenses will be identified such as training venue room hire, IT equipment and training materials. These costs will be obtained retrospectively from the research team immediately after training. Other training-related expenses will cover accommodation, travel and subsistence for trainers and experts by experience if not included in the stipend; translators and subsistence for translators; trainee stipend and subsistence for trainees if not covered in the stipend; driver time payment; petrol/diesel; refreshments; and catering expenses for the trainee and any other person accompanying the trainer or the trainee to assist with child care.

### Data analysis

Quantitative data will be assessed using generalised linear mixed models depending on the distribution of the outcome (continuous, binary). Descriptive statistics of quantitative outcome data (OSCE score, ASTAD, knowledge quiz) will be provided. The impact of the training on outcome measures will be analysed by comparing the OSCE scores, ASTAD and knowledge quiz at baseline, post-training and 3 months post-training. A three-level hierarchical model will be employed, and all time points will be included as repeated measures in the model to improve power and account for the clustering of observations at site levels.

Qualitative data will be analysed by thematic analysis [39] of the interview and focus group transcripts using the NVivo qualitative computer software. Coding and translation of illustrative accounts will be undertaken by research staff fluent in the language of the transcript and English so that a coding framework in English based on data from all sites can be created. A combined deductive and inductive approach will be applied to identify and compare themes across sites in terms of responses to the training methods and content, including the implementability of the knowledge and skills learned including barriers encountered.

For health economics analysis, the training cost will be summed and weighted by the number of individuals trained and the number of training sessions to derive a total cost per trainee and a total cost per session. Pooled and site-specific training costs will be reported. We will describe the total and component training costs presenting median, mean, standard deviations and ranges.

One-way sensitivity analyses will be undertaken to explore the sensitivity of the results to changes in assumptions.

## Discussion

Data from this study will inform decisions on the part of the research teams at each site regarding whether a trial of effectiveness and cost-effectiveness should be carried out. In the service of this decision, this study has several strengths in terms of the intervention and evaluation designs. The training draws upon the evidence bases for stigma reduction, health advocacy and medical education. In addition, the training is tailored from the outset to each site through the selection of locally relevant data from the situational analyses. Furthermore, the content, delivery methods and intensity of the training have been agreed upon through a consensus exercise with site research teams to enhance feasibility. Other strengths of the evaluation design include the use of a mixed methods design the use of a behavioural outcome measure in the form of the OSCE and the use of knowledge and attitude measures appropriate for the target population and the training. Key limitations are the uncertain generalisability of OSCE performance to routine care and our inability to assess effectiveness or cost-effectiveness in relation to patient outcomes at this stage. Finally, the existence of COVID-19 restrictions at several sites limited the extent of qualitative data collection for the situational analyses to inform the development of READ-MH.

The range of study sites in low- and middle-income settings will generate data on feasibility and acceptability likely to be transferable to other such settings. Although some single-site studies have been carried out with mental health professionals in middle-income countries [40, 41], to date, work in low-income countries on health care provider stigma has focused mainly on primary care [42] and medical students [43]. Furthermore, the aim of such studies, as reflected by their different choice of outcome measures, is stigma reduction rather than equipping health professionals as anti-stigma agents. Mental health professionals working in low- and middle-income settings are often keenly aware of their advocacy role and we hope this study will help to build their capacity to carry it out.

## Data Availability

The dataset resulting from this project will be available in the de-identified format on reasonable request to the first author. The manual and measures will also be available on reasonable request to the first author.

## Abbreviations

ASTAD: Attitudes to addressing stigma and discrimination scale
OSCE: Objective Structured Clinical Examination
READ-MH: Responding to Experienced and Anticipated Discrimination
